# Primary hyperoxaluria in adults and children: a nationwide cohort highlights a persistent diagnostic delay

**DOI:** 10.1093/ckj/sfae099

**Published:** 2024-04-03

**Authors:** Romain Pszczolinski, Cécile Acquaviva, Insaf Berrahal, Nathalie Biebuyck, Stéphane Burtey, Karine Clabault, Claire Dossier, Matthieu Guillet, Floriane Hemery, Emmanuel Letavernier, Caroline Rousset-Rouvière, Justine Bacchetta, Bruno Moulin

**Affiliations:** Service de néphrologie-dialyse-transplantation, Hôpitaux universitaires de Strasbourg, Strasbourg, France; Service de biochimie et biologie moléculaire, CHU de Lyon HCL – GH Est, Lyon, France; Service de néphrologie, CHU Dupuytren, Limoges, France; Service de néphrologie pédiatrique, Hôpital Necker-Enfants malades, Assistance Publique-Hôpitaux de Paris, Paris, France; Service de néphrologie et de transplantation rénale, Hôpital de la Conception, Assistance Publique-Hôpitaux de Marseille, Marseille, France; C2VN, Aix-Marseille Université/INSERM/INRAE, Marseille, France; Service de néphrologie, Hôpital Privé de l'Estuaire, Le Havre, France; Service de néphrologie pédiatrique, Hôpital Robert-Debré, Assistance Publique-Hôpitaux de Paris, Paris, France; Service de néphrologie, Hôpital Bicêtre, Assistance Publique-Hôpitaux de Paris, Le Kremlin-Bicêtre, France; Service de pédiatrie, CHU de Montpellier, Montpellier, France; Service d'Explorations fonctionnelles multidisciplinaires, Hôpital Tenon, Assistance Publique-Hôpitaux de Paris, Paris, France; Service de pédiatrie multidisciplinaire, Hôpital de la Timone, Assistance Publique-Hôpitaux de Marseille, Marseille, France; Service de néphrologie-rhumatologie-dermatologie pédiatriques, CHU de Lyon HCL – GH Est-Hôpital Femme Mère Enfant, Lyon, France; Service de néphrologie-dialyse-transplantation, Hôpitaux universitaires de Strasbourg, Strasbourg, France

**Keywords:** diagnostic delay, genetic testing, nephrolithiasis, orphan disease, primary hyperoxaluria

## Abstract

**Background:**

Primary hyperoxalurias (PH) are extremely rare genetic disorders characterized by clinical heterogeneity. Delay in diagnosing these conditions can have detrimental effects on patient outcomes. The primary objective of this study is to assess the current diagnostic delay for PH.

**Methods:**

This nationwide, observational and retrospective study included patients who received a genetic diagnosis of PH types 1, 2 and 3 between 1 January 2015 and 31 December 2019. Diagnostic delay was defined as the duration between the onset of symptoms and the time of genetic diagnosis.

**Results:**

A total of 52 patients (34 children and 18 adults) were included in the study, with 40 PH1 (77%), 3 PH2 (6%) and 9 PH3 (17%). At the time of diagnosis, 12 patients (23%) required dialysis. Among the PH1 patients, the predominant symptom at onset in adults was renal colic (79% of cases), whereas symptoms in children were more diverse (renal colic in 17% of cases). The diagnostic delay was significantly shorter in children compared with adults [median (interquartile range)]: 1.2 (0.1–3.0) versus 30 (17–36) years, respectively (*P *< .0001). RNA interference was utilized in 23 patients (58%). Five individuals (13%) underwent double liver–kidney transplantation, and five (13%) received isolated kidney transplantation, with lumasiran therapy in four patients. For PH2 and PH3 patients, the diagnostic delay ranges from 0 to 3 years, with renal colic as first symptom in 33% of cases.

**Conclusion:**

This extensive and recent cohort of PH underscores the considerable delay in diagnosing PH, particularly in adults, even in a country with a dedicated organization for enhancing the overall management of rare diseases. These findings reinforce the imperative for increased awareness among relevant specialties regarding the evaluation of urolithiasis.

KEY LEARNING POINTS **What was known**:

- Primary hyperoxalurias (PH) represent extremely rare genetic disorders characterized by the excessive production of oxalate, leading to urolithiasis, oxalate deposition in various tissues and ultimately kidney failure especially in type 1, whereas types 2 and 3 generally exhibit milder symptoms.
- Early diagnosis is crucial, as it enables prompt intervention, potential slowing down or halting disease progression, and enhancing patient outcomes.
- However, significant diagnostic delays can occur, likely attributable to the clinical heterogeneity and atypical presentations of PH.

**This study adds**:

- This study evaluate the current diagnostic delay for PH, through a recent, nationwide, observational and retrospective cohort.
- For PH1, in the cohort of 24 children included, the median diagnostic delay was 1.2 years, whereas it reached 30 years in the 16 adults.
- For PH2 and PH3 patients, the diagnostic delay ranged from 0 to 3 years in 12 patients.

**Potential impact:**

- These results underscore the considerable delay in diagnosing PH, particularly in adults.
- Consequently, these findings emphasize the crucial need for increased awareness of this disease among physicians who encounter cases of urolithiasis, to ensure timely referral of patients to specialized clinicians.

## INTRODUCTION

Primary hyperoxaluria (PH) is a rare recessive autosomal disorder with an estimated prevalence of 1–3 cases per million [1]. Three types of PH are described according to the mutated gene involved in glyoxylate metabolism. PH type 1 (PH1, MIM 259900) is caused by loss-of-function mutations of the alanine–glyoxylate aminotransferase (*AGXT*) gene; PH type 2 (PH2, MIM 260000) in the glyoxylate reductase/hydroxypyruvate reductase (*GRHPR*) gene; and PH type 3 (PH3, MIM 613616) in the 4-hydroxy-2-oxoglutarate aldolase 1 (*HOGA1*) gene [2]. However, the prevalence of PH expected by population genetic studies is much higher than in clinical cohorts [3], suggesting underdiagnosis.

Patients typically present with nephrolithiasis and/or nephrocalcinosis, which progress rapidly to kidney failure, at least in PH1 [4]. When renal function falls below 30 mL/min/1.73 m^2^, oxalate accumulation occurs in various tissues, including bones, eyes, vessels and heart, also known as ‘systemic oxalosis’, leading to diverse clinical manifestations and worsening global outcomes. However, the phenotypic presentation can vary significantly [5, 6], ranging from severe infantile oxalosis to milder symptoms in adulthood. The increasing identification of PH cases with less severe or atypical phenotypes, especially type 3, suggests potential underdiagnosis. Therefore, further characterization of phenotypes in newly diagnosed PH patients is necessary.

Patients with PH are frequently exposed to a significant diagnostic delay, resulting in poor outcomes [7, 8] and suboptimal management [9]. According to different studies [7, 8, 10, 11], 20%–57% of patients present with kidney failure at diagnosis. In addition, the diagnostic delay varies widely among individuals [8], suggesting a potential improvement in specific patient subgroups. Late diagnosis could be mainly attributed to atypical forms or a lack of awareness among physicians. The management of orphan diseases in France has been structured at the national level since 2004, with reference centres for rare renal diseases and the French network for rare kidney diseases (ORKID). Since 2017, this organization has been further extended at the European level through the European Reference Network for Rare Kidney Diseases (ERKNet). However, it remains unclear whether this structuration has fully addressed diagnostic delays in ultra-rare diseases.

Furthermore, RNA interference (RNAi) has emerged as a promising therapeutic approach for PH [12–17], to date at least in type 1. The possibility of performing isolated kidney transplantation combined with RNAi offers the hope of a suspensive treatment of this disease [18]. If long-term results confirm these findings, early diagnosis before the development of untreatable complications will be more crucial than ever.

Thus, the primary objective of this study is to evaluate the current diagnostic delay in recently diagnosed PH patients in France, to describe the underlying phenotypes and examine patients’ trajectories leading to diagnosis.

## MATERIALS AND METHODS

This work was a nationwide, multicentre, retrospective and observational study.

All patients who were diagnosed with PH types 1, 2 or 3 between 1 January 2015 and 31 December 2019 through genetic testing at the laboratory that received samples from all suspected cases in France were considered. The medical records available at various medical centres were reviewed.

Clinical parameters closest to the genetic diagnosis including age, gender, height, body weight, laboratory tests results and renal status were documented. The medical history, including symptoms at onset and the physicians involved in the follow-up before the genetic diagnosis, was recorded, as well as the clinical and biological evolution after genetic diagnosis. Any previous incorrect diagnosis were listed.

Patients were categorized as children if they were 18 years of age or younger. For non-dialysed patients, the estimated glomerular filtration rate (eGFR) at diagnosis was calculated using the Chronic Kidney Disease Epidemiology Collaboration equation (CKD-EPI) for adults and the 2009 Schwartz’ formula [19] for children. Kidney failure was defined as dependence on dialysis or kidney transplant.

Urinary oxalate levels were recorded when eGFR was above 30 mL/min/1.73 m^2^ and measured using enzymatic technique or gas chromatography mass spectrometry (GC-MS). GC-MS is more specific than the enzymatic technique, potentially causing variations between measurements. Serum oxalate levels, if available, were recorded when eGFR was ≤30 mL/min/1.73 m^2^ and measured by GC-MS with stable isotope dilution. Unpublished data from the Lyon laboratory indicated that serum oxalate and plasma oxalate were comparable. Radiographic examination reports were collected for all patients.

Diagnostic delay was defined as the difference between the age at onset of symptoms and the age at genetic diagnosis. Ages were recorded in years for adults and in months for children to ensure better accuracy. For patients who received a genetic diagnosis before the onset of initial symptoms through family screening or prenatal diagnosis, the diagnostic delay was considered as 0. Pyridoxine responsiveness was defined as a decline of urinary oxalate:creatinine ratio of >30% with pyridoxine therapy because data on urinary oxalate per 24 h were missing [20].

Genetic testing was initially conducted through classical sequential Sanger sequencing of the three genes for patients diagnosed in 2015 and 2016, and subsequently through massively parallel sequencing (MPS) allowing the simultaneous study of the three genes. Both methods allow for the detection of genetic variants located in exons and intron/exon boundaries. Given that Sanger sequencing cannot detect copy number variations, screening for large deletions or duplications in the *AGXT* gene was performed using multiplex ligation-dependent probe amplification (MLPA) testing. Since 2017, the MPS method allows to detect both copy number variations and single nucleotide variations. The performance of these methods was initially validated using control samples with known mutations.

The incidence rate was calculated by dividing the total number of cases by the sum of the French population on 1 January of each year (2015–19). Continuous variables are reported as medians (interquartile ranges, IQR) due to the non-Gaussian distribution of most variables. Non-parametric Mann–Whitney U tests were performed. Categorical variables are reported as numbers and percentages. The statistical analysis were conducted using R 3.1 (R Development Core Team, 2008) with GMRC Shiny Stat software (2017). A *P*-value <.05 was considered significant for all analyses.

This study received approval from the independent local ethics committee of the University Hospital of Strasbourg, during the session held on 22 December 2020, with the reference number CE-2020-205. In accordance with French law, non-opposition to participation was sought for all patients.

## RESULTS

A total of 62 patients had a genetic diagnosis of PH between 1 January 2015 and 31 December 2019, resulting in an incidence rate of 0.19 newly diagnosed patients per million per year in France. One patient refused to participate in the study, and data for nine patients were unavailable. Therefore, the analysis included 52 patients from 48 families across 27 centres in France. Thirty-four patients (65%) were children, and 18 (35%) were adults.

The main characteristics of patients are displayed in Table 1. The distribution of patients according to the underlying genotype was as follows: PH1 in 40 patients (77%), PH2 in 3 patients (6%) and PH3 in 9 patients (17%). Urolithiasis was reported in 40 patients (83%, 4 missing data), and nephrocalcinosis in 20 patients (40%, 2 missing data). The patients’ geographical origins were diverse: 15 (29%) from Western Europe, 17 (32%) from Maghreb, 13 (25%) from Eastern Europe, 2 (4%) from Asia and origin unknown for 5 patients (10%). The healthcare pathway varied significantly among patients. Before the genetic diagnosis, urologists were involved in the follow-up of 17 patients (32%), paediatricians in 12 children (35%) and nephrologists in 6 adult patients (33%).

**Table 1:** Patients’ characteristics.

	PH1 (*N* = 40)		PH2 (*N* = 3)		PH3 (*N* = 9)		
	Children	Adult	Children^b^	Adult^b^	Children	Adult^b^	Total
Number (% of all patients)	24 (46)	16 (31)	2 (4)	1 (2)	8 (15)	1 (2)	52
Age at diagnosis (years) [medians (IQR)]	2.2 (1.5–8.4)	41 (37–53)	0.3; 1.5	53	5.5 (2.8–14)	21	9 (2–36)
Female (%)	15 (63)	5 (31)	2	0	2 (25)	1	25 (48)
eGFR at diagnosis (mL/min/1.73 m^2^) (kidney failure excluded)	117 (68–130)	36 (24–59)	97; 100	90	120 (90–126)	125	102 (66–128)
Kidney failure at diagnosis (%)	4 (17)	8 (50)	0	0	0	0	12 (23)
Treatment of kidney failure at the last follow-up^a^ (%)							
Haemodialysis	1 (25)	5 (45)	0	0	0	0	6 (40)
Peritoneal dialysis	0	0	0	0	0	0	0 (0)
Kidney transplantation	3 (75)	6 (55)	0	0	0	0	9 (60)
Presence of consanguinity (8 MD) (%)	8 (42)	7 (50)	2	0	0	MD	17 (39)
Familial history of genetically confirmed PH (%)	3 (13)	5 (31)	2	0	3 (38)	0	13 (25)
With consanguinity (%)	3 (13)	2 (13)	2	0	0	MD	7 (13)
Familial history of urolithiasis without confirmed PH (%)	8 (33)	4 (25)	0	1	0	0	13 (25)
With consanguinity (%)	2 (8.3)	4 (25)	0	0	0	MD	6 (12)
Growth delay (6 MD) (%)	7 (29)	0	2	0	0	0	9 (20)
Urolithiasis (4 MD) (%)	15 (68)	13 (93)	2	1	8	1	40 (83)
Nephrocalcinosis (2 MD) (%)	13 (56)	7 (47)	0	0	0	0	20 (40)
Haematuria (gross or microscopic) (6 MD) (%)	6 (27)	4 (31)	0	1	2 (29)	0	13 (28)
Diagnostic delay (6 MD) (years) [medians (IQR)]	1.2 (0.1–3.0)	30 (17–36)	0; 1.3	35	1.0 (0.8–1.8)	MD	1.8 (0.5–14)

Values are medians (IQR) or numbers (percentages). Except for the first line, percentages are calculated on the basis of the number of patients in each subgroup. For percentages of 0 or 100, only the number of patients is indicated. Missing data (MD) are indicated in brackets.

a Three adult PH1 patients progressed to kidney failure after diagnosis.

b For groups with fewer than three patients, values are directly indicated, and separated by semi colons.

eGFR calculated by 2009 Schwartz’ formula for children, and CKD-EPI for adults.

**Figure 1:**
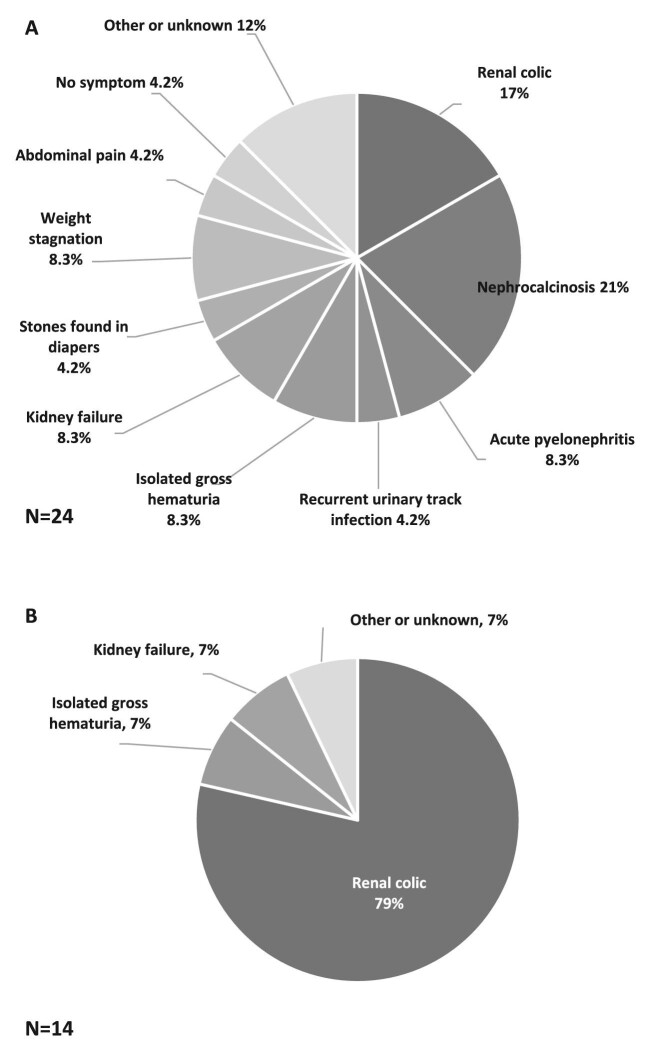
Distribution of symptoms at onset of PH1 in children (**A**) and in adults (**B**). Two missing data in adults.

**Figure 2:**
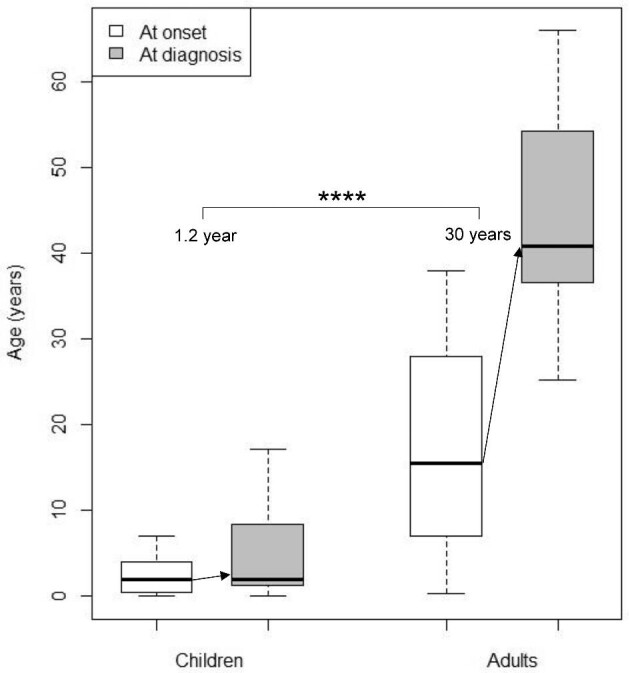
Age at onset (white boxes) and age at genetic diagnosis (grey boxes) of PH1 in children (*N* = 23) and adults (*N* = 13). Diagnostic delay is indicated with arrows. ****P < .0001.

For PH1 patients, symptoms at onset were highly heterogeneous in children but less diverse in adults (Fig. 1). Among children, the ‘classical’ presentation with renal colic or nephrocalcinosis occurred in nine patients (38%), while non-specific symptoms were observed: weight stagnation, abdominal pain, stones in diapers and urinary tract infection. The diagnostic delay for PH1 patients was 1.2 (0.1–3.0) years in children, and 30 (17–36) years in adults (*P *< .0001) (Fig. 2). Age at onset of symptoms was not recorded for three patients (one child and two adults). One adult patient was excluded from this analysis since specific treatment was initiated long before the genetic diagnosis, suggesting an earlier actual diagnosis. Thus, the diagnostic delay was calculated for 36 patients (23 children and 13 adults). Two patients had a genetic diagnosis before birth through familial screening when a case of PH was identified in the family. Patients with kidney failure at diagnosis had a longer median diagnostic delay, but the difference was not statistically significant [median diagnostic delay 13 (4.4–32) versus 1.5 (0.3–12) years, *P *= .06], probably due to a lack of statistical power. The diagnostic delay was significantly longer in patients with renal colic at onset [19 (11–35) years] than other symptoms [1.2 (0.2–2.9) years] (*P*-value <.001). However, upon adjustment for age groups, the difference was not significant. This result primarily underscores the difference in diagnostic delay between children (experiencing a high diversity of symptoms at onset) and adults (mainly presenting with renal colic). The diagnostic delay was not associated with sex, consanguinity, geographical origin, presence of nephrocalcinosis or presence of haematuria. Three patients received incorrect diagnoses before the genetic confirmation of PH, including enteric hyperoxaluria, distal renal tubular acidosis and coeliac disease. At the time of diagnosis of PH1, 12 patients (30%) had kidney failure, and three adult patients progressed to kidney failure after the genetic diagnosis, with a median follow-up since genetic diagnosis of 46 (32–62) months. Two patients died after diagnosis: one child at 15 months from cardiac failure after pulmonary septic shock during dialysis, and one adult at 38 years from an unknown cause. Extra-renal manifestations described in the medical records were observed only in PH1 patients: three patients with multiple bone deposits, two patients with neurological symptoms (proximal muscle deficit and epilepsy), two patients with cutaneous deposits, one patient with digital gangrene and one patient with macular crystals. All these patients had kidney failure at diagnosis. Cardiac, rheumatologic and endocrine manifestations were not found, although their systematic assessment was rarely mentioned in the medical records. Nine PH1 patients (23%) had no medical follow-up before the genetic diagnosis. Among them, seven were children: two experienced abrupt infantile oxalosis before 4 months of age, and four had an abrupt but less severe onset before 5 years of age, without requiring prior medical follow-up. One patient was diagnosed at 17 years of age despite symptoms present for 6 years, and did not receive specific follow-up. The remaining two patients were foreign adults who arrived in France after initiating haemodialysis. The genetic diagnosis was made at the beginning of their management in France. The absence of medical follow-up before the genetic diagnosis was not significantly associated with the risk of kidney failure at diagnosis (*P *= .21), but was linked to a shorter diagnostic delay [median diagnostic delay 0.2 (0.0–0.7) versus 3.0 (1.0–17.1) years, *P *< .05]. This result is likely explained by the most severe forms of PH (namely infantile oxalosis) resulting in short diagnostic delay without previous medical follow-up.

Among the three patients with PH2, two were children from the same family. The diagnostic delay was 0 and 1.3 years, with the first symptoms (stones in diapers) occurring at 7 and 3 months, respectively. The adult patient experienced first symptom (isolated gross hematuria) at the age of 2 years, and the diagnosis was suspected around the age of 18 years based on biochemical values, leading to standard treatment for PH. The genetic diagnosis was made 35 years later. Among the nine patients with PH3 (eight children and one adult), the diagnostic delay was 1.0 (0.8–1.8) years for seven children with first symptoms after 2015. The diagnostic delay cannot be reported for the last child and the adult, because they experienced first symptoms before the recognition of the type 3 as a distinct type in 2010 [21]. Symptoms at onset were renal colic in four children, isolated gross haematuria in two children, and acute pyelonephritis in two children and the adult. All patients with PH2 or PH3 had an eGFR >60 mL/min/1.73 m^2^ at diagnosis, and none of them had extra-renal manifestations. None of these patients developed kidney failure.

Among the whole cohort, liver biopsies were performed in three patients, with one patient undergoing liver biopsy in 2006 to measure alanine:glyoxylate aminotransferase activity, which was normal. Genetic assessment eventually confirmed the diagnosis of PH3. The other two liver biopsies were conducted before the patients arrived in France for unknown reasons, and both cases showed moderate portal fibrosis. Three patients underwent kidney biopsies for evaluating kidney failure, all of which revealed the presence of whewellite crystals.

Biological parameters were inconstantly evaluated in the medical charts. Urinary oxalate:creatinine ratio at diagnosis was not available for seven patients. The results of urinary oxalate by age groups are summarized in Table 2. Urinary oxalate per 24 h was available for 10 patients, with a median value of 1.37 (0.84–1.79) mmol per 24 h. Serum oxalate at diagnosis was measured in 21 patients—14 children and 7 adults. Six patients (three children and three adults) had an eGFR ≤30 mL/min/1.73 m^2^. All other patients (11 children, 4 adults) had an eGFR >50 mL/min/1.73 m^2^. Recently published guidelines [17] recommend not to assess plasma oxalate in patients with eGFR above 30 mL/min/1.73 m^2^. Urinary and serum glycolate were assessed in too few patients to be reported.

**Table 2:** Urinary oxalate:creatinine ratios at diagnosis by age class and type of PH, and reference ranges described by Barratt *et al.* [22].

	Urinary oxalate:creatinine ratio at diagnosis (µmol/mmol)			
Age class (years)	PH1	PH2	PH3	Reference range [22]
<1 year (*N* = 6)	470 (190–490)	260	/	15–260
1 to <5 years (*N* = 16)	480 (140–670)	288	220 (200–250)	11–120
5 to <13 years (*N* = 5)	200 (160–260)	/	117	60–150
13 to 18 years (*N* = 4)	130	/	25; 71; 110	2–80
>18 years (*N* = 14)	150 (140–250)	135	92	2–80

Values are medians (IQR). For groups with fewer than four patients, values of urinary:creatinine ratios are directly indicated, and separated by semi colons. Groups with no patient are indicated with ‘/’.

Numbers of patients in each age class are indicated in brackets. Seven missing data.

Among patients with PH1, 19 different pathogenic variants were identified. The most frequent variant was c.731T>C (p.Ile244Thr), accounting for 31% of PH1 alleles, followed by c.508G>A (p.Gly170Arg), accounting for 29% of PH1 alleles. *AGXT* pathogenic variants clearly associated with pyridoxine responsiveness, namely c.508G>A (p.Gly170Arg) and c.454T>A (p.Phe152Ile) [9, 20, 23], were found in homozygous state in 10 patients (25%) and in compound heterozygous state with another pathogenic variant in 5 patients (13%). Another *AGXT* pathogenic variant that may be associated with pyridoxine responsiveness, c.731T>C (p.Ile244Th), was identified in homozygous state in 12 patients. Genotypes of PH2 and PH3 patients are summarized in Table 3.

**Table 3:** Detailed genotypes of patients with PH2 and PH3.

Patients	Allele 1 variant	Allele 2 variant
PH2		
Patient 1^a^	c.494G>A (p.Gly165Asp)	c.494G>A (p.Gly165Asp)
Patient 2^a^	c.494G>A (p.Gly165Asp)	c.494G>A (p.Gly165Asp)
Patient 3	c.370C>T (p. Arg124Cys)	c.454dup (p.Thr152fs)
PH3		
Patient 1^b^	c.700+5G>T (/)	c.700+5G>T (/)
Patient 2^b^	c.700+5G>T (/)	c.700+5G>T (/)
Patient 3^b^	c.700+5G>T (/)	c.700+5G>T (/)
Patient 4	c.700+5G>T (/)	c.700+5G>T (/)
Patient 5	c.700+5G>T (/)	c.700+5G>T (/)
Patient 6	c.208C>T (p.Arg70X)	c.700+5G>T (/)
Patient 7	c.208C>T (p.Arg70X)	c.208C>T (p.Arg70X)
Patient 8	c.668C>T (p.Ser223Leu)	c.700+5G>T (/)
Patient 9	c.110G>T (p.Gly37Val)	c.502T>C (p.Tyr168His)

^a,b^Denote siblings. No missing data. “/” indicates the absence of an amino acid modification in the protein, due to intronic variation.

The treatments received after genetic diagnosis in patients with PH1 are described in Table 4. In total, 23 patients (58%) received RNAi therapy. Five patients (13%) underwent liver–kidney transplantation, and 5 patients (13%) received an isolated kidney transplantation. Among them, four patients received an isolated kidney graft between 2020 and 2022 under lumasiran, as previously reported for three of these patients [18], and maintained a functional graft at the last follow-up. The remaining patient received a kidney graft abroad in 2016 for kidney failure, before the diagnosis of PH. The kidney graft was lost within a month, and the patient returned to haemodialysis. No PH2 or PH3 patients received RNAi therapy. All three PH2 patients and one PH3 patient received treatment with stiripentol.

**Table 4:** Detailed specific management for patients with PH1.

	Children (*N* = 24)	Adults (*N* = 16)	Total (*N* = 40)
Age at initiation of treatment (years) [median (IQR)]	2 (1–6)	45 (28–57)	7.5 (2–34)
Treatment (%)			
Crystallization inhibitor	23 (96)	13 (81)	36 (90)
RNA interference	16 (67)	7 (44)	23 (58)
Lumasiran	15 (63)	7 (44)	22 (55)
Nedosiran	1 (4)	0 (0)	1 (3)
Stiripentol	4 (17)	2 (13)	6 (15)
Treatment by pyridoxine (%)	23 (96)	13 (81)	36 (90)
Patients with 2 pyridoxine-responsiveness mutations^a^	5 (21)	5 (31)	10 (25)
Patients eventually responsive to pyridoxine	3 (13)	0 (0)	3 (8)
Isolated kidney transplantation under RNA interference (%)	2 (8)	2 (13)	4 (10)
Isolated kidney transplantation without RNA interference (%)	0 (0)	1 (6)	1 (3)
Liver and kidney transplantation (%)	1 (4)	4 (25)	5 (13)
Age at kidney transplantation (years) [median (IQR)]	18 (3–19)^b^	35 (27–45)	27 (20–38)

Values are medians (IQR) or numbers (percentages). No missing data.

a Considered pyridoxine-responsiveness mutations were c.508G>A (p.Gly170Arg) and c.454T>A (p.Phe152Ile).

b Figure in brackets indicate minimum and maximum because there are only three patients in this group.

## DISCUSSION

To our knowledge, this study represents the first nationwide approach to comprehensively describe a large cohort of PH1-2-3 patients in France. The key finding is the extremely long diagnostic delay in adults, despite the presence of reference centres for rare diseases in our country since 2004.

In children, the diagnostic delay appears reasonable and roughly comparable to other studies. However, adult patients experienced longer diagnostic delays. The definition of diagnostic delay was consistent across studies. A previous cohort conducted in the Netherlands [8] reported a median diagnostic delay of 5.5 years in 29 adults and 0.1 year in 50 children. Similarly, the German experience revealed that diagnostic delays were <1 year in two-thirds of 65 patients, while ranging from 1 to 31 years in 16 patients [24]. It is worth noting that these studies relied on surveys distributed to (paediatric) nephrologists and did not encompass a nationwide evaluation. Such study design could introduce a selection bias towards physicians with a higher interest in urolithiasis. In an Italian cohort, the median diagnostic delay was observed to be 3.2 years in 95 patients [11]. Despite the wide range of symptoms at onset of paediatric PH, paediatricians appear sufficiently aware of the diagnosis. In fact, in France and other European countries, it is standard practice to refer children with kidney stones to paediatric nephrology centres for evaluation [25]. In contrast, physicians appeared to be less inclined to consider the diagnosis of PH in adults, despite the relatively homogeneous symptoms at onset. Kidney stones in adults are often regarded as trivial, indicating a need for greater physician awareness when patients present with urolithiasis, especially if the stones are recurrent, bilateral, occur at a young age or are accompanied by consanguinity or nephrocalcinosis. In France, guidelines for managing urolithiasis [26] call for a minimal evaluation starting from the first episode. It is of high importance to conduct a comprehensive evaluation in children or young adults, or patients with bilateral or recurrent stones, nephrocalcinosis or a history of consanguinity. In addition, the study highlighted a lack of comprehensive evaluation for urinary oxalate and its metabolites, compared with the most recent European guidelines [17], which specifically recommend assessing 24-h urinary oxalate as soon as the patient acquired urinary autonomy, and performing at least two urine assessments to establish hyperoxaluria. A more complete biochemical assessment could facilitate early diagnosis, and comparisons between different studies. The diagnostic delay observed in our study for patients with PH2 was remarkably short, aligning with the results reported in the OxalEurope registry. The registry documented a delay of 1.8 years in 101 patients [27], with a median age of 3.2 years at the onset of symptoms. However, patients with PH3 exhibited longer diagnostic delays, likely due to a less severe phenotype. The OxalEurope registry also reported a diagnostic delay of 4 years in 95 patients with PH3 [28], with a median age at first symptoms of 1.8 years.

The proportion of PH3 cases in our study (17%) was higher compared with previously published studies. This difference can be attributed to familial screening (three patients were siblings) and the recent recognition of PH3 as a distinct type [21], leading to underestimation in previous cohorts. This explanation is supported by the apparent increase in the diagnosis of PH3 over time. In 2005 and 2009, ‘non-PH1/PH2’ forms accounted for 7% and 5% of all cases, respectively [1, 7], which increased to 11% in 2015 [3] and 12% in 2022 [29].

This study revealed a large diversity of symptoms at onset, including paucisymptomatic and atypical presentations, increasing the risk of diagnostic failure. These results differ from foreign cohorts, which described less diverse symptoms at onset [8, 11], such as high frequencies of nephrolithiasis and nephrocalcinosis. Nevertheless, in our cohort, the occurrence of extra-renal manifestations was infrequent compared with the Dutch cohort [30]. Several factors may explain these findings, including increased awareness of the disease, the nationwide design of our study, and a recent rise in the diagnosis of milder or atypical forms. The former are more likely to present with fewer extra-renal manifestations, while the latter may exhibit more diverse symptoms. This is supported by the fact that our study identified 52 new cases within a span of only 5 years, whereas previous cohorts covered longer periods, suggesting either a lower incidence of diagnosis or a selection bias resulting from the use of surveys that prevent exhaustive data collection. As specific genetic diagnosis are conducted in a single centre in France, our study provides a nearly exhaustive representation of cases. We have also contacted other laboratories in France that are capable of diagnosing PH through panel gene or exome analysis. None of them reported any PH diagnosis during the study period. Furthermore, a study by Sas *et al*. [31] reported comparable PH severity among patients diagnosed following clinical manifestation and those identified through familial screening. However, the latter group exhibited fewer stones at the time of diagnosis. These findings emphasize the importance of early diagnosis and treatment, even though clinical presentation is poor.

The study also identified a lower frequency of pathogenic variants associated with pyridoxine-responsive phenotypes than previously reported [9, 20, 32], suggesting a greater diversity of genotypes.

The study's strengths include its quasi-exhaustive diagnosis of PH in France, confirmed genetic diagnosis for all patients, and the relatively short and recent study period. However, there are limitations to consider. First, genetic tests are highly reliable, but it is important to note that molecular testing does not allow the detection of variations located in promoters, enhancers or deep introns. Second, the retrospetive design of the study prevents analysis of risk factors or prognostic factors. Third, the number of missing data limits the conclusions that can be drawn. Lastly, longer follow-up is necessary to determine the impact of diagnostic delay on prognosis, including dialysis requirement and the influence of new treatments.

In conclusion, this large and recent cohort highlights a significant delay in the diagnosis of PH, especially among adults. This delay is primarily attributed to the lack of diagnosis rather than diagnostic errors, even in a country with a dedicated organization focused on enhancing the overall management of rare diseases. While the prognosis of PH holds the potential for significant improvements in the coming years, it remains a severe and highly diverse condition that requires early diagnosis. Even though urologists and paediatric urologists play a key role in managing these patients, there is a critical need for them to take a more active role in the diagnosis, as proposed in the most recent European and French guidelines [17]. However, it is imperative to enhance awareness of these ultra-rare diseases on a global scale, particularly considering the availability of focused therapeutic options.

## Data Availability

The data underlying this article will be shared on reasonable request to the corresponding author.

## References

[1] Hoppe B, Beck BB, Milliner DS. The primary hyperoxalurias. Kidney Int 2009;75:1264–71. 10.1038/ki.2009.32 19225556 PMC4577278

[2] Mandrile G, Beck B, Acquaviva C et al. Genetic assessment in primary hyperoxaluria: why it matters. Pediatr Nephrol 2023;38:625–34. 10.1007/s00467-022-05613-2 35695965 PMC9842587

[3] Hopp K, Cogal AG, Bergstralh EJ et al. Phenotype-genotype correlations and estimated carrier frequencies of primary hyperoxaluria. J Am Soc Nephrol 2015;26:2559–70. 10.1681/ASN.2014070698 25644115 PMC4587693

[4] Bouzidi H, Majdoub A, Daudon M et al. Hyperoxalurie primitive : une revue de la littérature. Nephrol Ther 2016;12:431–6. 27372182 10.1016/j.nephro.2016.03.005

[5] Cochat P, Rumsby G. Primary hyperoxaluria. N Engl J Med 2013;369:649–58. 10.1056/NEJMra1301564 23944302

[6] Strauss SB, Waltuch T, Bivin W et al. Primary hyperoxaluria: spectrum of clinical and imaging findings. Pediatr Radiol 2017;47:96–103. 10.1007/s00247-016-3723-7 27844104

[7] Lieske JC, Monico CG, Holmes WS et al. International Registry for Primary Hyperoxaluria. Am J Nephrol 2005;25:290–6. 10.1159/000086360 15961949

[8] van der Hoeven SM, van Woerden CS, Groothoff JW. Primary hyperoxaluria Type 1, a too often missed diagnosis and potentially treatable cause of end-stage renal disease in adults: results of the Dutch cohort. Nephrol Dial Transplant 2012;27:3855–62. 10.1093/ndt/gfs320 22844106

[9] van Woerden CS, Groothoff JW, Wijburg FA et al. Clinical implications of mutation analysis in primary hyperoxaluria type 1. Kidney Int 2004;66:746–52. 10.1111/j.1523-1755.2004.00796.x 15253729

[10] Harambat J, van Stralen KJ, Espinosa L et al. Characteristics and outcomes of children with primary oxalosis requiring renal replacement therapy. Clin J Am Soc Nephrol 2012;7:458–65. 10.2215/CJN.07430711 22223608 PMC3302673

[11] Mandrile G, Pelle A, Sciannameo V et al. Primary hyperoxaluria in Italy: the past 30 years and the near future of a (not so) rare disease. J Nephrol 2022;35:841–50. 10.1007/s40620-022-01258-4 35218550 PMC8995259

[12] Hayes W, Sas DJ, Magen D et al. Efficacy and safety of lumasiran for infants and young children with primary hyperoxaluria type 1: 12-month analysis of the phase 3 ILLUMINATE-B trial. Pediatr Nephrol 2023;**38**:1075–86. 10.1007/s00467-022-05684-1 PMC9925547 35913563

[13] Michael M, Groothoff JW, Shasha-Lavsky H et al. Lumasiran for advanced primary hyperoxaluria type 1: phase 3 ILLUMINATE-C Trial. Am J Kidney Dis 2023;81:145–155.e1. 10.1053/j.ajkd.2022.05.012 35843439

[14] Garrelfs SF, Frishberg Y, Hulton SA et al. Lumasiran, an RNAi therapeutic for primary hyperoxaluria type 1. N Engl J Med 2021;384:1216–26. 10.1056/NEJMoa2021712 33789010

[15] Hoppe B, Koch A, Cochat P et al. Safety, pharmacodynamics, and exposure-response modeling results from a first-in-human phase 1 study of nedosiran (PHYOX1) in primary hyperoxaluria. Kidney Int 2022;101:626–34. 10.1016/j.kint.2021.08.015 34481803

[16] Baum MA, Langman C, Cochat P et al. PHYOX2: a pivotal randomized study of nedosiran in primary hyperoxaluria type 1 or 2. Kidney Int 2023;103:207–17. 10.1016/j.kint.2022.07.025 36007597

[17] Groothoff JW, Metry E, Deesker L et al. Clinical practice recommendations for primary hyperoxaluria: an expert consensus statement from ERKNet and OxalEurope. Nat Rev Nephrol 2023;19:194–211. 10.1038/s41581-022-00661-1 36604599

[18] Sellier-Leclerc A-L, Metry E, Clave S et al. Isolated kidney transplantation under lumasiran therapy in primary hyperoxaluria type 1: a report of 5 cases. Nephrol Dial Transplant 2023;38:517–21. 36307929 10.1093/ndt/gfac295

[19] Schwartz GJ, Muñoz A, Schneider MF et al. New equations to estimate GFR in children with CKD. J Am Soc Nephrol 2009;20:629–37. 10.1681/ASN.2008030287 19158356 PMC2653687

[20] Monico CG, Rossetti S, Olson JB et al. Pyridoxine effect in type I primary hyperoxaluria is associated with the most common mutant allele. Kidney Int 2005;67:1704–9. 10.1111/j.1523-1755.2005.00267.x 15840016

[21] Belostotsky R, Seboun E, Idelson GH et al. Mutations in DHDPSL are responsible for primary hyperoxaluria type III. Am Hum Genet 2010;87:392–9. 10.1016/j.ajhg.2010.07.023 PMC2933339 20797690

[22] Barratt TM, Kasidas GP, Murdoch I et al. Urinary oxalate and glycolate excretion and plasma oxalate concentration. Arch Dis Child 1991;66:501–3. 10.1136/adc.66.4.501 2031609 PMC1792981

[23] Williams EL, Acquaviva C, Amoroso A et al. Primary hyperoxaluria type 1: update and additional mutation analysis of the AGXT gene. Hum Mutat 2009;30:910–7. 10.1002/humu.21021 19479957

[24] Hoppe B, Latta K, von Schnakenburg C et al. Primary hyperoxaluria—the German experience. Am J Nephrol 2005;25:276–81. 10.1159/000086358 15961947

[25] Rauturier C, Machon C, Demède D et al. Composition of urinary stones in children: clinical and metabolic determinants in a French tertiary care center. Eur J Pediatr 2021;180:3555–63. 10.1007/s00431-021-04151-7 34165592

[26] Saussine C, Lechevallier E, Traxer O. Urolithiasis and guidelines. Prog Urol 2008;18:841–3. 10.1016/j.purol.2008.09.043 19033040

[27] Garrelfs SF, Rumsby G, Peters-Sengers H et al. Patients with primary hyperoxaluria type 2 have significant morbidity and require careful follow-up. Kidney Int 2019;96:1389–99. 10.1016/j.kint.2019.08.018 31685312

[28] Martin-Higueras C, Garrelfs SF, Groothoff JW et al. A report from the European Hyperoxaluria Consortium (OxalEurope) Registry on a large cohort of patients with primary hyperoxaluria type 3. Kidney Int 2021;100:621–35. 10.1016/j.kint.2021.03.031 33865885

[29] Singh P, Viehman JK, Mehta RA et al. Clinical characterization of primary hyperoxaluria type 3 in comparison with types 1 and 2. Nephrol Dial Transplant 2022;37:869–75. 10.1093/ndt/gfab027 33543760 PMC9214566

[30] van Woerden CS, Groothoff JW, Wanders RJA et al. Primary hyperoxaluria type 1 in The Netherlands: prevalence and outcome. Nephrol Dial Transplant 2003;18:273–9. 10.1093/ndt/18.2.273 12543880

[31] Sas DJ, Enders FT, Mehta RA et al. Clinical features of genetically confirmed patients with primary hyperoxaluria identified by clinical indication versus familial screening. Kidney Int 2020;97:786–92. 10.1016/j.kint.2019.11.023 32093915 PMC7175669

[32] Harambat J, Fargue S, Acquaviva C et al. Genotype-phenotype correlation in primary hyperoxaluria type 1: the p.Gly170Arg AGXT mutation is associated with a better outcome. Kidney Int 2010;77:443–9. 10.1038/ki.2009.435 20016466

